# Endocardial ablation of a suspected mid-myocardial or epicardial ventricular tachycardia using a lattice tip ablation catheter: A case report

**DOI:** 10.1016/j.hrcr.2026.02.007

**Published:** 2026-02-16

**Authors:** Prateeth Pati, George Shaw, Joshua Silverstein, Amit Thosani

**Affiliations:** Department of Cardiac Electrophysiology, Allegheny Health Network, Pittsburgh, Pennsylvania

**Keywords:** Ventricular tachycardia, Ablation, Epicardial, Cardiomyopathy, Pulsed field ablation

**Key Teaching Points**

• Ablation of mid-myocardial and epicardial ventricular tachycardia (VT) is frequently complicated and may require several attempts at ablation.
• Pulsed-field ablation (PFA) in concert with radiofrequency ablation (RF) using newer dual-energy catheter systems is a feasible method to allow targeting of mid-myocardial and epicardial VT via an endocardial approach.
• PFA has complementary effects to RF technology, allowing for deeper and more comprehensive lesion sets when used together.

## Introduction

Epicardial ventricular tachycardia (VT) is disproportionately common in non-ischemic cardiomyopathy and is a potential cause for recurrent VT after ablation. Epicardial ablation has improved outcomes in such cases but is associated with risk including coronary artery and phrenic nerve injury, pericardial effusion or tamponade, and epicardial fat limiting ablation lesion depth, amongst other risks.^1^ Pulsed field ablation (PFA) technologies, either stand alone or in combination with radiofrequency ablation, may offer strategies for mid-myocardial and epicardial ablation via an endocardial approach. We describe a case of successful ablation of a mid-myocardial or epicardial, peri-mitral VT with endocardial-only ablation using a lattice tip catheter (Sphere 9, Medtronic).

## Case Report

A 66-year-old man with heterozygous Filamin C (*FLNC*) mutation-positive nonischemic dilated cardiomyopathy, left ventricular ejection fraction 30%–35%, cardiac arrest, and secondary prevention implantable cardioverter-defibrillator (ICD) presented with recurrent VT despite 2 prior ablation procedures.

In October 2018, he presented with VT storm treated with 12 ICD shocks. He underwent an electrophysiology (EP) study where 3 VT morphologies were induced. Initial activation mapping localized early sites to the basal inferolateral wall. Pace mapping (97% match) was performed to localize the area of interest, and ablation was then performed to encompass this region. Following ablation, programmed stimulation from the left ventricle (LV) (600-300-250-250) induced VT #2, which was activation mapped to the lateral perimitral region. Long duration ablation here resulted in gradual slowing and termination of the VT. Following this, programmed stimulation was again performed (600-300-250-250 ms), triggering VT #3. This VT was activated and pace-mapped to the LV outflow tract, just underneath the right coronary cusp. Following this, programmed stimulation was performed from the LV on up to 5 mcg/min of isoproterenol, with triple extra-stimuli at a 400 ms drive cycle length down to ventricular effective refractory period (400-300-290-240 ms). No further sustained arrhythmias were noted. He was discharged the next day on his pre-admission mexiletine and sotalol. Amiodarone was not used as he had failed to tolerate this in the past because of gastrointestinal upset. Of note, he was diagnosed with post-traumatic stress disorder related to his multiple ICD shocks.

The patient did well with routine follow-ups until May 2022, when he was hospitalized for Influenza A after returning from a trip to Africa. In this context, he experienced multiple runs of monomorphic VT treated with multiple anti-tachycardia pacing runs and a single ICD shock. Viral panel revealed he was positive for Influenza A. He was treated for this with Tamiflu, after which he had no further recurrence of VT. His anti-arrhythmic medications and guideline-directed medical therapy were continued as before on discharge, given his VT was felt to be secondary to his acute illness, which had resolved. He did well with routine follow-up after this point.

**Figure 1:**
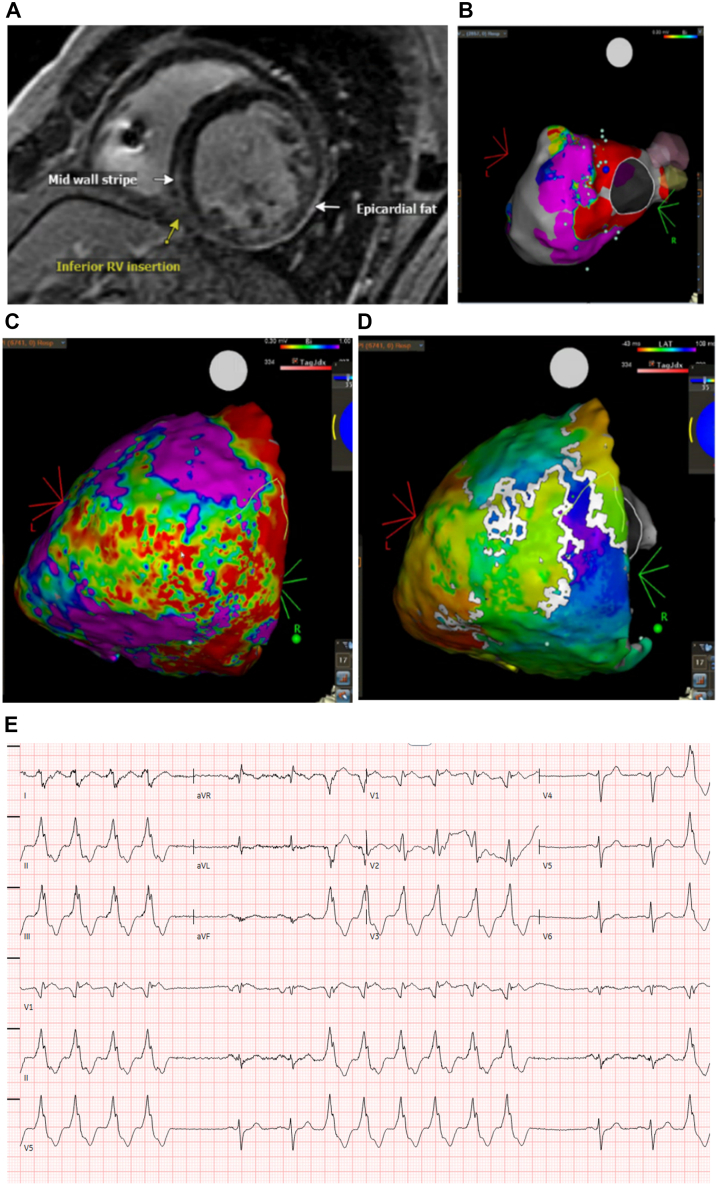
**A:** cMRI with prominent mid-myocardial LGE, consistent with nonischemic cardiomyopathy. **B:** Extensive endocardial perimitral voltage abnormality. **C, D:** Epicardial voltage map with large area of perimitral voltage abnormality and latest activation at lateral mitral annulus. **E:** ECG with recurrent salvos of monomorphic VT. cMRI = cardiac magnetic resonance imaging; ECG = electrocardiogram; LGE = late gadolinium enhancement; VT = ventricular tachycardia.

In June 2025, after multiple ICD shocks for recurrent monomorphic VT (Figure 1), he underwent endocardial-epicardial mapping and radiofrequency ablation. Pre-procedural cardiac magnetic resonance imaging showed dilated LV with left ventricular ejection fraction 42%, and perimitral mid-myocardial and epicardial late gadolinium enhancement (Figure 1). Epicardial mapping revealed a large area of perimitral epicardial voltage abnormality with concordant endocardial perimitral low-voltage areas (Figure 1, Supplemental Videos 1 and 2).

**Figure 2:**
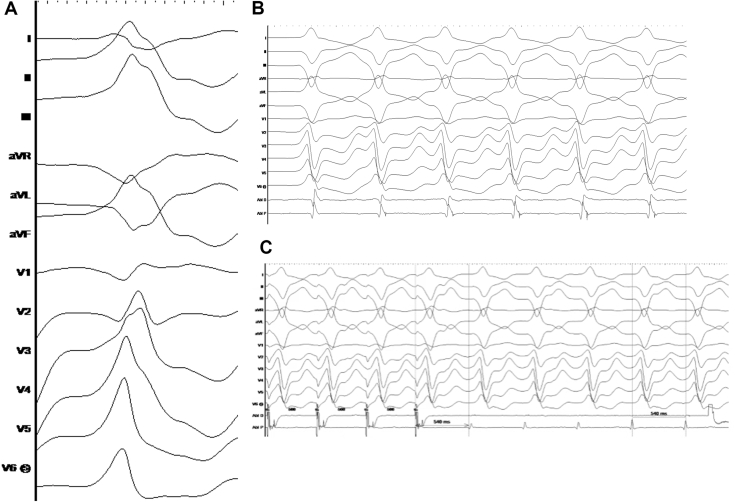
**A:** Spontaneous PVC(s) noted during isoproterenol infusion (left). **B:** Sustained VT induced during substrate modification and entrainment mapped to lateral mitral region (above). **C:** Concealed entrainment mapping demonstrated by pacing at the perimitral epicardium. PVC = premature ventricular complex; VT = ventricular tachycardia.

VT was non-inducible despite extensive programmed stimulation and isoproterenol infusion, although spontaneous premature ventricular complexes (PVC) that were consistent with the morphology of the clinical VT were noted (Figure 2). Coronary angiography was performed, and the circumflex and obtuse marginals were carefully avoided during subsequent radiofrequency (RF) ablation (QDot Micro, Johnson and Johnson Medtech). The frequent PVC was mapped and ablated at 3 o’clock on the epicardial mitral annulus, followed by extensive endocardial and epicardial perimitral substrate ablation. During substrate modification, sustained VT was induced (Figure 2); this tachycardia was entrainment mapped to the perimitral epicardium at approximately the 4 o’clock position. VT terminated during ablation and additional perimitral epicardial substrate ablation was performed. The patient was monitored for 2 days after ablation with no further VT. Amiodarone was started but not tolerated, and he was discharged on sotalol and mexiletine.

**Figure 3:**
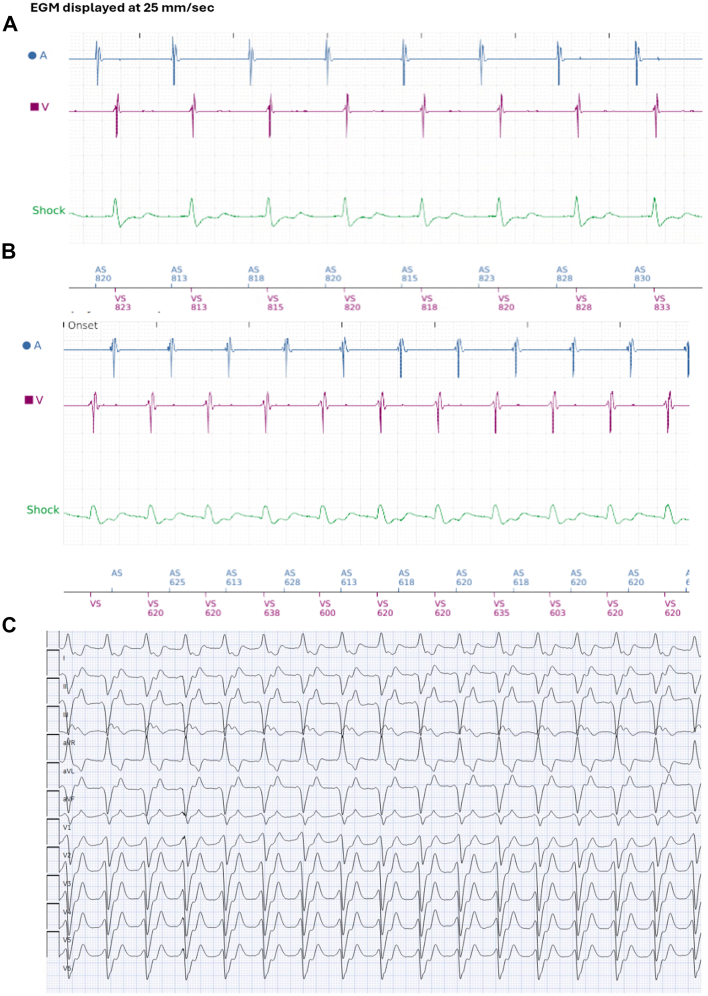
**A:** Device captured normal sinus rhythm. **B:** Device captured ventricular rhythm with 1:1 VA conduction. **C:** 12-lead ECG of slow ventricular rhythm. ECG = electrocardiogram; VA = ventriculo-atrial.

2 months later, the patient developed exertional dyspnea. He was found to be in a slow ventricular rhythm with 1:1 ventriculo-atrial (VA) conduction (Figure 3). He was hemodynamically stable with this rhythm, but was profoundly symptomatic and requested to be seen urgently in clinic. After a discussion of all available options, he elected for urgent direct-current cardioversion, which was successful in terminating the ventricular rhythm, and his symptoms. A repeat EP study was performed. Baseline ventricular rhythm with cycle length of 630 ms was present. Mechanical PVCs from retrograde ablation catheter advancement into the LV terminated the rhythm, which could unfortunately not be reinduced. No convincing endocardial pace map for the ventricular rhythm was found, suggesting an epicardial exit.

Unfortunately, the patient’s ventricular rhythm recurred shortly after and was persistent. He was hesitant to undergo repeat epicardial ablation; compassionate use approval was granted by the Food and Drug Administration and the local institutional review board for endocardial ablation attempt using a 9-mm lattice tip catheter capable of performing both radiofrequency ablation and PFA. This process required a letter explaining treatment strategy and failure of prior therapies including prior ablation and antiarrhythmic drugs from the treating physician and an unaffiliated physician be sent to both the local institutional review board, device company, and the Food and Drug Administration, followed by patient consent once approved by all regulatory bodies. No special exemption was needed for atrial parameters.

At the definitive procedure, the same ventricular rhythm as previously cardioverted and pace terminated was confirmed. Left femoral arterial access was obtained and endocardial LV voltage mapping was performed without any difficulty crossing the aortic valve. Trans-septal access as an alternative to retrograde access was considered, but given that the ease of aortic valve crossing and sufficient accessibility to the intended site of ablation was demonstrated during the prior ablation attempt, a decision was made to pursue aortic retrograde access. No special technique was necessary for crossing of the aortic valve. Infrequent ectopy was noted during left ventricular (LV) mapping, and the ventricular rhythm was sustained during the mapping portion of the procedure. Multiple entrainment attempts from the endocardial LV demonstrated fusion with long post-pacing interval, consistent with a non-endocardial exit.

**Figure 4:**
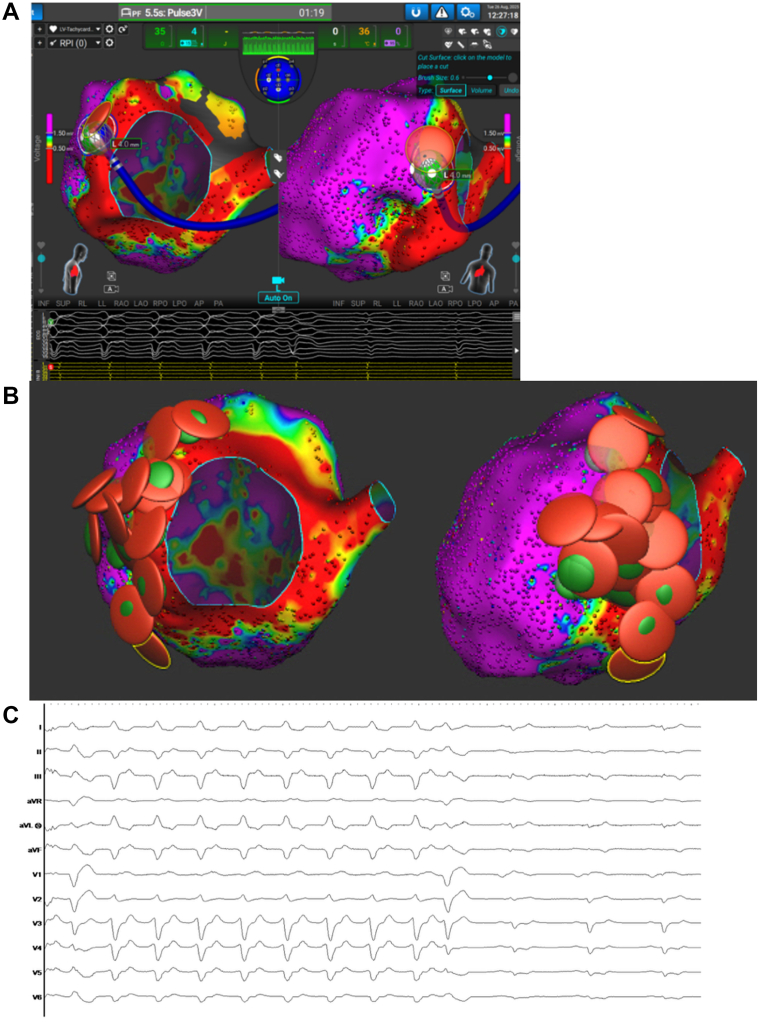
**A:** VT termination site. **B:** Ablation map with red denoting RF lesion and green denoting PF lesion. **C:** VT rhythm termination during RF application. PF = pulsed field; RF = radiofrequency; VT = ventricular tachycardia.

VT was abruptly terminated approximately 8 seconds into the initial RF application (temperature-controlled irrigated with target surface temperature of 60°C, 30 s duration) at approximately 2 o’clock on the endocardial mitral annulus (Figure 4). Multiple stacked RF and PF (biphasic monopolar delivery) lesions were then delivered surrounding this site for substrate ablation. RF was first administered using the above settings, immediately followed by PF application to achieve greater lesion penetration into mid-myocardial and epicardial regions. This RF-PF stacking approach was performed broadly across the lateral perimitral region for scar homogenization.

Interestingly, the electrocardiogram (ECG) pattern prior to the planned ablation did not correspond to the location of our definitive lesion. Given the relatively widespread nature of the patient’s ventricular scarring, we had planned to perform a broad RF-PF stacking strategy across the lateral perimitral region and did not expect VT termination to occur as expeditiously as it did. However, the seeming disparity between prior rhythm captured on ECG and termination in the lab could be explained by the globally heterogenous nature of the patient’s scar tissue.

## Discussion

In non-ischemic cardiomyopathy, epicardial and mid-myocardial substrate is common and frequently leads to failure of endocardial ablation alone.^1^, ^2^, ^3^, ^4^ Combined endo-epicardial or epicardial-only approaches are often necessary in such cases to successfully identify and ablate the clinical VT. However, pericardial access and ablation close to critical structures, such as the coronary arteries and phrenic nerve pose a significant risk with epicardial ablation.^1^ The broader footprint and electroporation field characteristics of PFA and combined RF-PFA systems may increase the likelihood of reaching such targets without the need for pericardial access in certain cases.

Preclinical data suggest that sequential, colocalized RF-PF lesion ‘stacking’ increased lesion depth and width compared with either modality alone, supporting its use when targeting suspected mid-myocardial or epicardial substrates.^5^ Biophysically, RF and PFA appear complementary. RF creates a central thermal coagulative core, whereas PFA adds a surrounding nonthermal electroporation injury zone. This partnership appears to expand lesion volume and depth, an effect supported by histology showing a thermal core with a transitional PF rim in swine ventricles.^5^

Multiple case series have demonstrated successful VT or PVC elimination using PFA catheters. Newer large footprint, dual-energy catheters such as the Sphere-9 system enable rapid mapping and energy toggling and have demonstrated acute procedural success in small cohorts and in individual cases, as in ours. Additionally, we subjectively noted a lower burden of ventricular ectopy with this catheter compared with traditional focal RF and multielectrode mapping catheters during LV mapping.

A recent report described the successful elimination of an epicardial ischemic scar-related VT using endocardial-only PFA, establishing proof of concept that epicardial circuits may be terminated from the endocardium in certain cases.^6^ Our case adds to this early experience, achieving acute VT termination and post ablation non-inducibility without the need for pericardial access in a nonischemic cardiomyopathy with peri-mitral VT. Larger, systemic evaluations are needed to determine the frequency with which suspected mid-myocardial/epicardial targets can be reached with endocardial ablation and whether specific structural, imaging, or electroanatomic features may predict success.

## Conclusion

In this case, an integrated mapping-ablation approach with a lattice tip catheter enabled successful endocardial treatment of an epicardial VT that had failed multiple prior ablations. To our knowledge, this represents one of the earliest reported cases of endocardial therapy resolving a mid-myocardial/epicardial VT circuit with this platform, extending an emerging literature.^6^

## Disclosures

The authors have no conflicts of interest to disclose.

## Footnotes

Appendix Supplementary data associated with this article can be found in the online version at https://doi.org/10.1016/j.hrcr.2026.02.007.
