# Supplementary material for: Endocardial ablation of a suspected mid-myocardial or epicardial ventricular tachycardia using a lattice tip ablation catheter: A case report
Source: HeartRhythm Case Rep. 2026 Feb 16;12(5):560–5. doi: 10.1016/j.hrcr.2026.02.007 (PMC13198268; doi:10.1016/j.hrcr.2026.02.007)
Supplement: Supplementary Video Legends [file mmc3.docx]

Video legends

Video 1

Epicardial voltage activation map

Video 2

Endocardial voltage activation map
